# Modeling partial lockdowns in multiplex networks using partition strategies

**DOI:** 10.1007/s41109-021-00366-7

**Published:** 2021-04-01

**Authors:** Adrià Plazas, Irene Malvestio, Michele Starnini, Albert Díaz-Guilera

**Affiliations:** 1 Departament de Física de la Matèria Condensada, Universitat de Barcelona, 08028 Barcelona, Catalonia Spain; 2 Universitat de Barcelona Institute of Complex Systems (UBICS), Universitat de Barcelona, 08028 Barcelona, Catalonia Spain; 3 ISI Foundation, 10126 Turin, Italy

**Keywords:** NPIs modelling, SARS-CoV-2 virus, Multiplex networks, Epidemic processes

## Abstract

National stay-at-home orders, or lockdowns, were imposed in several countries to drastically reduce the social interactions mainly responsible for the transmission of the SARS-CoV-2 virus. Despite being essential to slow down the COVID-19 pandemic, these containment measures are associated with an economic burden. In this work, we propose a network approach to model the implementation of a partial lockdown, breaking the society into disconnected components, or partitions. Our model is composed by two main ingredients: a multiplex network representing human contacts within different contexts, formed by a Household layer, a Work layer, and a Social layer including generic social interactions, and a Susceptible-Infected-Recovered process that mimics the epidemic spreading. We compare different partition strategies, with a twofold aim: reducing the epidemic outbreak and minimizing the economic cost associated to the partial lockdown. We also show that the inclusion of unconstrained social interactions dramatically increases the epidemic spreading, while different kinds of restrictions on social interactions help in keeping the benefices of the network partition.

**Supplementary Information:**

The online version contains supplementary material available at 10.1007/s41109-021-00366-7.

## Introduction

The current COVID-19 pandemic left already more than two million deaths around the world and this number will increase in the near future (Dong et al. 2020). As a response, almost all countries implemented unprecedented measures to restrict individual mobility and promote social distancing. Starting by mid-March, several governments adopted a number of Non-Pharmaceutical Interventions (NPIs), whose severity rapidly increased in time: starting from school and university closures, large social gatherings avoidance, closure of non-essential activities, and finally a national stay-at-home order, or lockdown (Hsiang et al. 2020). The aim of these NPIs was to reduce and possibly interrupt the transmission of the SARS-CoV-2 virus. National lockdowns have demonstrated very effective in slowing down the spread of the COVID-19 epidemic (Flaxman et al. 2020; Walker et al. 2020), as the time-varying reproduction number of the epidemic—representing the mean number of secondary infections generated by one primary infected individual, over the course of an epidemic (Liu et al. 2018)—started to significantly drop few days after their implementation (You et al. 2020; Starnini et al. 2020). Despite being essential to contain the pandemic, these measures deeply affected the state of the economy, triggering a major world recession.^1^ For this reason, drastic NPIs such as national lockdowns have been adopted only for a limited time span. This apparent trade-off between public health and economy sparkled a heated debate regarding the optimal duration and intensity of lockdowns.

The ultimate goal of all NPIs is to decrease the number, duration, and frequency of social contacts among individuals, so to reduce the probability of virus transmission. The unfolding of social interactions can be represented by social networks, where nodes represent individuals and links stand for interactions (Jackson 2010). Network science has demonstrated to be a crucial tool to understand, model, and predict phenomena of social dynamics (Newman 2010; Castellano et al. 2009). The theoretical framework of network science has been recently enriched by two key concepts: Multi-layer networks (Boccaletti et al. 2014; Aleta et al. 2020), whose edges belong to different layers, representing different kinds of interactions; and temporal networks, whose edges appear and disappear in time, representing interactions switching on and off with given characteristic time scales (Holme and Saramäki 2012; Holme 2015). Both concepts have proved very useful for a deeper understanding of the dynamical processes on top of real networks, such as epidemic spreading (Lambiotte et al. 2013; De Domenico et al. 2016; Starnini et al. 2017; Estrada 2020).

Within a network perspective, the implementation of lockdowns can be effectively considered as link removal processes, in which nodes represent individuals and links represent their social interactions operated in different contexts, e.g. “at work”, “home”, or “school”. At the same time, the unfolding of the COVID-19 pandemics can be effectively represented as the spreading of an epidemic process on such networks (Pastor-Satorras et al. 2015). The aim of this study is to evaluate the effect on the pandemics of different (partial) lockdown strategies, all based on splitting the society into disconnected components, uniquely identified by a “color”. In network science, this translates into removing links. Here, we assume that these links are removed with respect to social interactions occurring at workplace, since other options (e.g. splitting households) are less feasible. This is associated, however, to an economic cost. In this work, we compare different strategies aimed at reducing such economic loss, while at the same time controlling the epidemic spread.

On general grounds, it is obvious that the more stringent the NPIs to halt the disease spreading, the larger the damage to the economy. This compromise between reducing the burden on the health care system and allowing the preservation of economic activities has been object of some recent works. For instance, the authors of Ref. de Vlas and Coffeng (2020) proposed a strategy to achieve herd immunity at the national level by releasing restrictive measures in specific localized areas, while favoring mobility of severe patients into other areas with less incidence. From a completely different perspective, other authors Meidan et al. (2021) proposed an alternating quarantine strategy for the whole population, which implies a 50% cut in the economic capacity while the effects in the epidemic spreading are encouraging. The comparative predicted effect of this alternating strategy clearly outperforms other measures of half-quarantine that consider a fixed half, either in time or in the population.

The structure of the paper we present here is as follows. In “Modelling social interactions as a multiplex network” section we describe the social network that models the substrate responsible for the virus transmission. In “Strategies for network partition” section we propose different partition strategies, which mainly correspond to different possible partial lockdowns. In “Epidemic spreading” section we show the results of numerical simulations of the epidemic spreading, while in “Effect of social interactions” section we address the effects of the inclusion of social interactions. Finally, the last section is devoted to conclusions.

## Modelling social interactions as a multiplex network

The spreading of a disease like COVID-19 requires close contacts during certain time. For instance, along a normal working day, people engage in different social interactions that can potentially infect other people. These contacts are usually modelled by networks, where different activities can be represented as different layers of a multiplex network (Kivelä et al. 2014). Multiplex networks have a long tradition in the modeling of epidemic spreading. Just to cite a few examples, in Chen et al. (2020), the multiplex network refers to alternative traffic routes between locations; in Soriano-Paños et al. (2018), the nodes are metapopulations and the different layers correspond to different social levels, or in Granell et al. (2013), one layer corresponds to the spreading of the epidemics and the other to the spreading of the awareness of the disease. Finally, several papers work on settings where the two layers correspond to different pathogens that can eventually trigger interactions (Wu and Chen 2020) or even multilayers corresponding to different agents, and therefore, contagions among nodes in different layers must also be considered (Cozzo et al. 2013; Wei et al. 2016).

For the sake of simplicity, here we consider individuals to have three kinds of interactions, similarly to what is proposed in Aleta et al. (2020), which are represented by three layers: Household, Work, and Social. According to the multiplex construction, individuals are the same across layers. Work and Household layers represent the strongest and well characterized fixed sets of connections in our daily life. A third Social layer is introduced to take into account the random and time-dependent social interactions that represent activities such as shopping, using public transportation, going to the gym, meeting friends, and so on. However, in our model the Social Layer takes into account the context of pandemics and containment of social interactions. For that reason the daily interactions in the Social Layer are modelled with a constant and low average connectivity, as we will see in “Effect of social interactions” section. We will show that if connections are dynamical, i.e. they change from day to day, the epidemic outbreak is larger than for constant connections, i.e. connections within the same pairs of nodes.

In our model, all individuals who work in the same company or department (from now on, workplace) are connected in a clique (a complete subgraph) in the Work layer, which is disconnected from cliques corresponding to other companies. A fraction of individuals is considered to work from home (or unemployed), thus being represented as isolated nodes in the Work layer, because they do not have close contact to other working people. On the other hand, as proposed in Aleta et al. (2020), we introduce household interactions by means of cliques, of smaller size, within the household layer. Note that the introduction of different types of interactions is not restricted to multiplex networks, but this construction permits to do it in a very intuitive way. In other works instead, networks are constructed with different structures, cliques, representing household interactions, together with some out of the clique links, representing all sort of work and/or social interactions, generating networks with a high degree of clustering (Ball et al. 2010; Britton et al. 2008; Mistry et al. 2021).

**Fig. 1:**
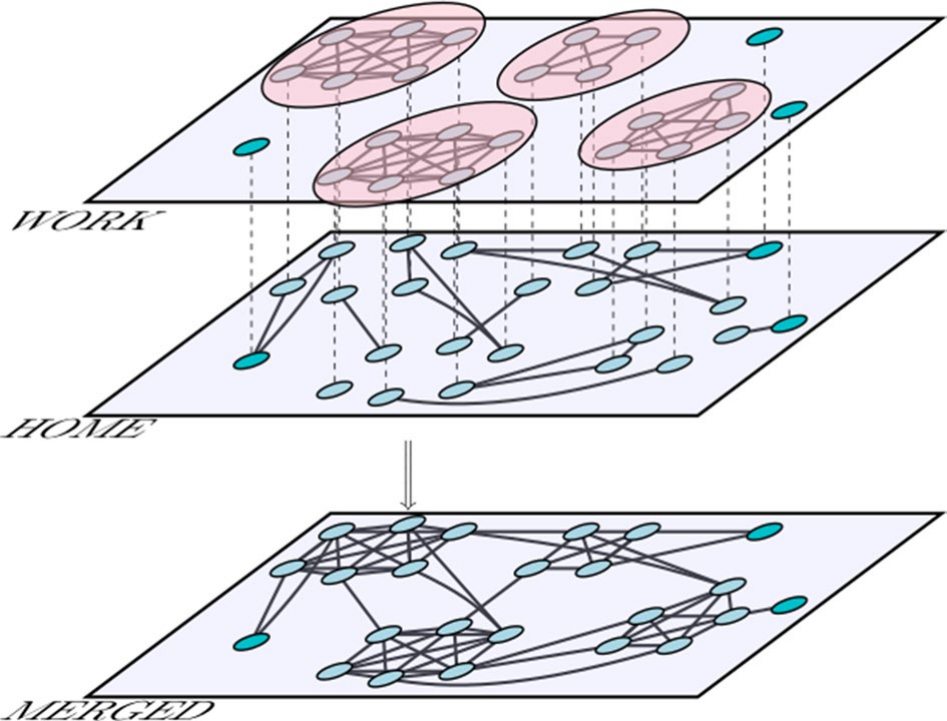
Multiplex construction of the two basic and fixed sets of connections. Top layer corresponds to the Work construction formed by cliques of relative large size. Bottom layer corresponds to the Household construction formed by cliques of very small size. Dashed lines are drawn just to recall that the nodes in the two layers correspond to the same individual. Merging these two layers into a single one results in a larger component, as shown in the single merged layer in the bottom of the panel

Individuals can also interact in the Social layer, according to different hypotheses described in “Effect of social interactions” section. Although Work and Household layers are formed by isolated cliques, it is the superposition of all the layers what generates a large connected component where a disease can easily spread. This basic multiplex construction (excluding the Social layer) is visualized in Fig. 1. This will be our initial setting that can be changed by containment measures. A connected component is not initially observed in any of the layers, but if layers are merged, a very large connected component (not necessarily spanning the whole network) shows up. Intuitively, this connected component shows how the epidemics can spread and reach most of the population.

Here, we focus on modelling a typical urban area, in which social interactions are more frequent and disease transmission easier. Note that the interplay between several urban and rural areas could be modelled through the adoption of a meta-population structure (Colizza and Vespignani 2008). We set a working scenario with the following distribution of household sizes: 1 member 0.38, 2 : 0.38, 3: 0.14, 4: 0.08, 5: 0.015, 6: 0.005. This data has been taken from the empirical distribution of the city of Barcelona.^2^ The distribution of workplace size is assumed to be a Gaussian with mean 15 and standard deviation 5. Other choices for the household and workplace size distribution are shown in the Additional file 1: Sect. I of the Supplementary Material. We also consider that $25\%$ of the population is not part of any workplace (these people either work from home or are unemployed). Notice that we consider that the maximum possible number of people who work from home is included already in this group. In the following simulations, the size of the network is set to $N = 5000$.

## Strategies for network partition

The aim of this study is to evaluate different (partial) lockdown strategies, which consist in splitting the society into disconnected components, identified by a “color”. The goal of this procedure is to make barriers to stop the disease spreading. We first assign colors to the workplaces. As individuals live in households, it can happen that individuals of the same household have different colors assigned. This situation would produce a conflict, namely a possible path of transmission to other companies, and hence to other families and so on, if there are infected individuals. In order to cut these potential paths of disease transmission, we solve the conflict by removing the node(s) from the Work layer and assigning all household members the same color. This has, however, an associated economic cost: individuals removed from their workplace cannot work from home (since we have already included the maximum percentage of work from home in the model). The aim of the strategies is to minimize the number of conflicts in order to reduce the economic loss but balancing it with a low disease transmission rate.

A strategy consists in assigning one color $C_j$ to each node $j=1, \dots , N$, from a set $\{c_i$, $i = 1, \dots , N_c \}$, where $N_c$ is the total number of colors. $N_c$ is a free parameter of the model; its limiting values are 1, corresponding to the original merged network which is the most fragile case in terms of the epidemic spread, and the number of workplaces, which is a complete segregation representing the worst economic scenario.

For the color assignment, we would ideally like to reach two (opposite) objectives: From one side, we would like to limit the fraction of conflicts ($\chi$); from the other side, we want to make sure that the assignment is as effective as possible in limiting the spreading of the virus. The effectiveness of a network segregation in slowing down the spreading is related to the size distribution of its components. This is because the larger a component is the more vulnerable to the disease (Newman 2002). We propose two magnitudes to quantify this vulnerability. On the one hand, the fraction of nodes that are part of the largest connected component of the network, *G*. On the other hand, we propose the entropy of the color distribution, which measures how much the color distribution is homogeneous. If $p_{c_i}$ is the fraction of nodes in the network with color $c_i$, the normalized entropy $S\left( \{ p_{c_i}\}\right)$ of the color distribution is:

$$\begin{aligned} S\left( \{ p_{c_i}\}\right) = \frac{1}{\log N_c^{-1}} \sum _{i = 1}^{N_c} p_{c_i} \log p_{c_i}. \end{aligned}$$

We consider four strategies for the color assignment, with a fixed number of colors $N_c$. They can be divided in strategies based on *Aggregation* (*Random, Maximal and Minimal*) and a strategy based on the *Segregation* of the network. In the *Aggregation* based strategies, we start by assigning a different color to each workplace (corresponding to a clique). Then we iteratively merge pairs of colors, until we reach the desired total number of colors $N_c$. We finally solve conflicts (household members with different colors) by removing nodes from the Work layer. In the *Segregation* strategy instead, we start with all nodes having assigned the same color. Then, we remove nodes from the Work layer, up to obtaining a number of disconnected components equal to the desired number of colors.

***Random Aggregation***: We start by assigning a different color to each workplace. The number of colors is reduced by *randomly* merging pairs of workplaces, without any rule. In this way we completely break up the network, but at the cost of a high number of removed nodes.
***Maximal Aggregation***: Similar to the previous one: we start with a different color for each workplace; then we iterate by merging colors together. The first color $c_i$ to be merged is selected at random, while, differently from the random case, the second color $c_j \ne c_i$ corresponds to (one of) the most popular colors among the neighbours node of $c_j$. That is the reason why we call this strategy *Maximal*, we choose the color with the maximum frequency. After some colors are joined together, they easily become more popular than others, and they are frequently found as neighbors of other colors. Therefore, the most popular is likely to attach more colors and so it becomes even more popular. Thus, this approach will tend to produce one large cluster and many small ones.
***Minimal Aggregation***: This strategy works similarly but it aims at a more homogeneous color distribution, thus it merges first those colors assigned to the smallest set of nodes. The algorithm is the same as for *Maximal Aggregation*, with the difference that the first color $c_k$ to be merged is not selected at random, but as the rarest between the colors available. That is the reason why we call this strategy *Minimal*, we choose the color with the minimum frequency. This difference proves to be sufficient to get a much uniform color distribution compared to the *Maximal Aggregation*.
***Segregation***: In this strategy, differently from the *Aggregation* based strategies, we start with all nodes having assigned the same color. Then, we remove nodes from the Work layer in descendent order of their betweenness centrality (Freeman 1977), up to obtaining a number of disconnected components equal to the desired number of colors $N_c$. The betweenness centrality is computed on the network corresponding to the collapse of the two layers (Sole-Ribalta et al. 2014), and it is recomputed after each node removal.

**Fig. 2:**
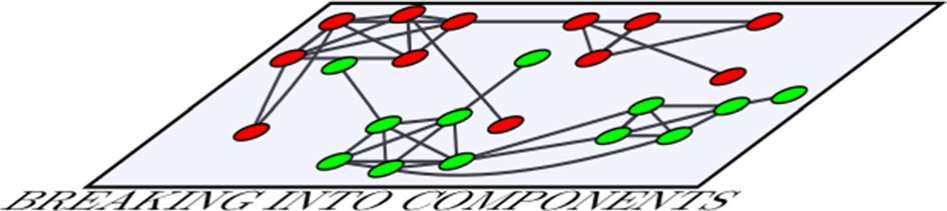
The merged network that gives rise to a single connected component in Fig. 1 is now splitted into two components (red and green) by removing some work links

Figure 2 shows the multiplex network introduced in Fig. 1 as broken into two independent components. Sizes of the components are very similar and the node removal from the Work layer has been minimized. Individuals who work from home have assigned the color that corresponds to their household.

**Fig. 3:**
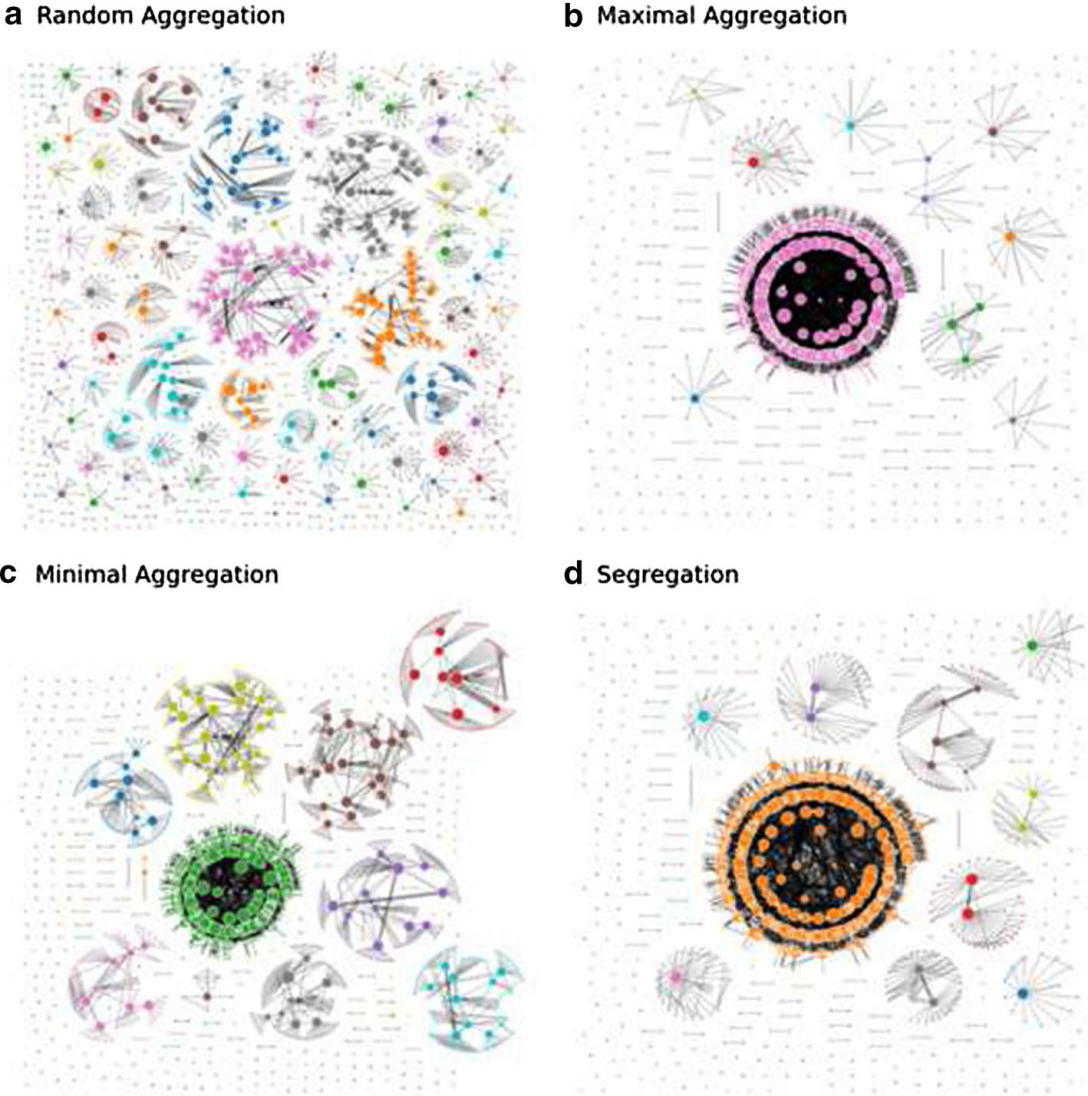
Network of workplaces. Each node represents a different workplace, whose size is proportional to the number of people working in that workplace. If the workers of two workplaces are part of the same household, the workplaces are connected by a link. The link width is proportional to the number of common housemates. The smallest dots represent people who work from home or do not work (workplace of size 1). The nodes are colored according to the strategy (different for each panel from **a**–**d**). The number of colors chosen is $N_c = 10$

Figure 3 shows a representation of the network partitioned according to different strategies. The nodes represent the workplace cliques, or alternatively, single individuals who work from home. The links connect two nodes if there is at least one pair of members sharing a household. The colors identify separate connected components in the networks. We can see how the *Random Aggregation* strategy has a more uniform color distribution but clearly disconnects the network into many small components, while at the other extreme the *Maximal Aggregation* and *Segregation* strategies show the predominance of one color corresponding to one big connected cluster. The *Minimal Aggregation* strategy is somehow a balance between these two behaviours.

**Fig. 4:**
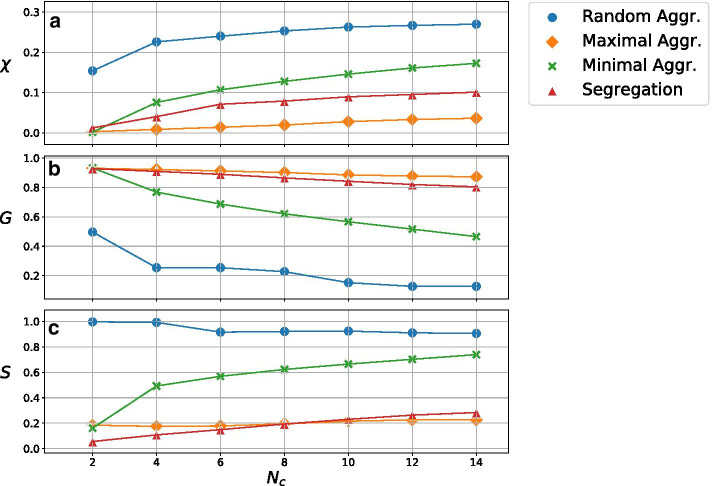
Fraction of conflicts $\chi$ (**a**), relative size of the biggest connected component *G* (**b**), and entropy of the color distribution *S* (**c**) as a function of the number of colors in the network $N_c$, for the different containment strategies

In Fig. 4 we compare the performance of the different strategies for the network partition. For each strategy, we show the fraction (with respect to the whole population) of conflicts in the network $\chi$, the relative size of the largest connected component *G*, and the entropy *S* of the color distribution, as a function of the number of colors $N_c$. As expected, an increasing number of colors gives a more disconnected network (smaller *G*) and a more homogeneous color distribution (higher *S*), while the number of conflicts $\chi$ increases. For the *Random Aggregation* and *Maximal Aggregation* strategies, the entropy is almost constant for the interval of colors considered. Coherently to what we notice in Fig. 3, the *Random Aggregation* method is the one that gives the best performance in term of breaking the connectivity of the network (smallest size of the largest connected component) and has a more homogeneous color distribution, as assessed by the entropy. But the *Random Aggregation* method is also the one with the highest percentage of conflicts, as expected. *Maximal Aggregation* and *Segregation* have the opposite trend: a low number of conflicts, but a very high proportion of the nodes are still connected, while the color distribution is not very homogeneous. Finally, the *Minimal Aggregation* method can provide an intermediate number of conflicts, while being able to disrupt the network connectivity with a relatively low number of colors ($N_c \approx 10$).

**Fig. 5:**
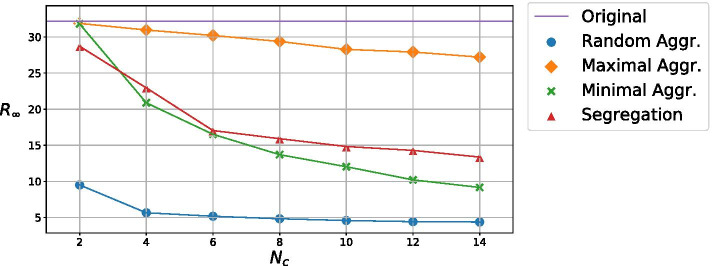
Final fraction of recovered agents $R_{\infty }$ for the considered strategies as a function of the number of colors $N_c$. The purple line represent the corresponding value for the original case with no color. Error bars are not plotted for being negligible

We also study the dependence of these results on the workplace and family size distribution (see of Additional file 1: Sect. I, Figs. S1 and S2). As a matter of fact, societies having different structural characteristics, and implementing different distributions allow us to identify which characteristics have implications for our results. We therefore consider company size distributions with different average and variance, and we conclude that the influence of the company size distribution is minimal (Fig. S1). For the household size distribution instead, we included more and less skewed version compared to the original one, to consider cases in which is more or less common to have a higher number of people sharing the same house. We found that the household size distribution, indeed, has an influence on our results (Additional file 1Fig. S2): the inclusion of household with more members gives rise to a higher number of conflicts, and hence more economic cost, and larger connected components.

**Fig. 6:**
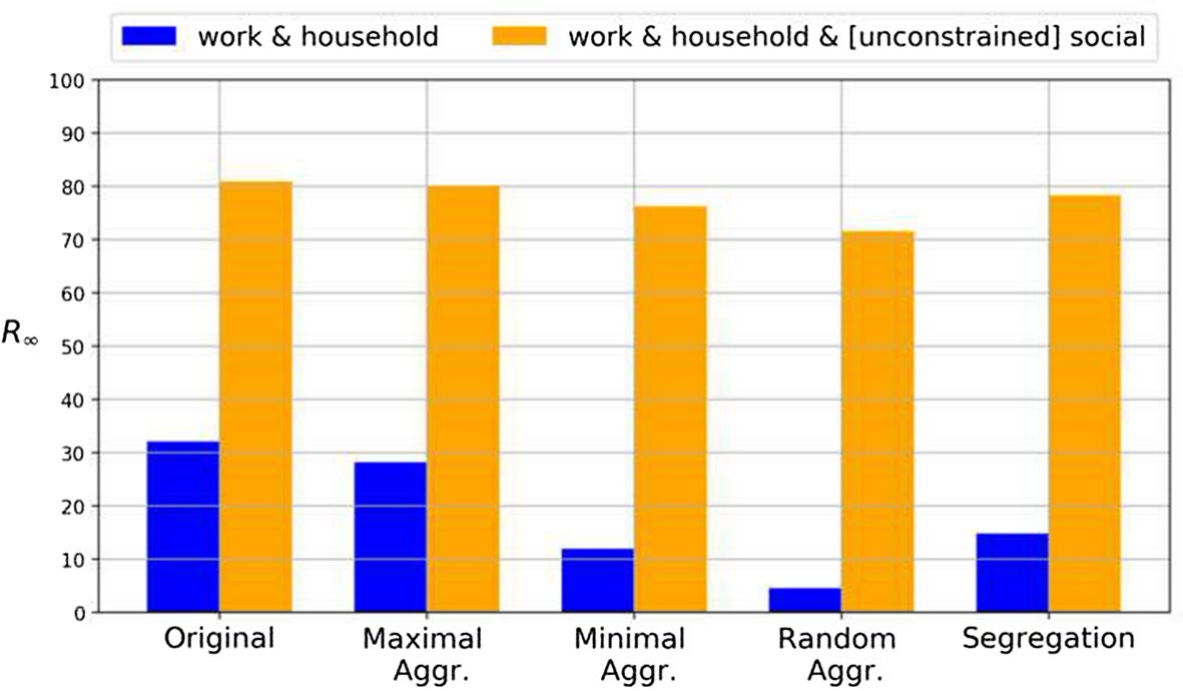
Fraction of recovered agents, $R_{\infty }$, for different strategies. Here we compare a multiplex with only Work and Household layers (without Social layer) and a multiplex which includes also an unconstrained Social layer. The color strategies are for the case of 10 colors. Error bars are not plotted for being negligible

The theoretical problem of finding a division in colors of the network that leads to the segregation of the largest connected component reminds the work of Kundu and Manna on colored percolation (Kundu and Manna 2017). In their work though the links are possible only between nodes of different colors, while in our case links are possible within the same colors. Future theoretical work can focus on the difference between these two scenarios.

## Epidemic spreading

In order to assess the effectiveness of the lockdown strategies presented in the previous section, we simulate the Susceptible-Infected-Recovered (SIR) model (Kermack and McKendric 1927) on the multiplex network. Briefly, in the SIR model, agents can be in one of three states: Susceptible, Infected, or Recovered. Upon contact with an Infected agent, a Susceptible agent becomes Infected with probability $\beta$ at each time step. Infected agents spontaneously become Recovered with probability $\gamma$, at each time step. Therefore, at each time step, infected agents may infect susceptible agents in contact with them, until no more infected agents are present in the population and the infection dies out. The final fraction of recovered agents, $R_\infty$, indicates how many agents became infected over the course of the infection and thus measures its intensity: the larger $R_\infty$, the more the disease spread in the population.

**Fig. 7:**
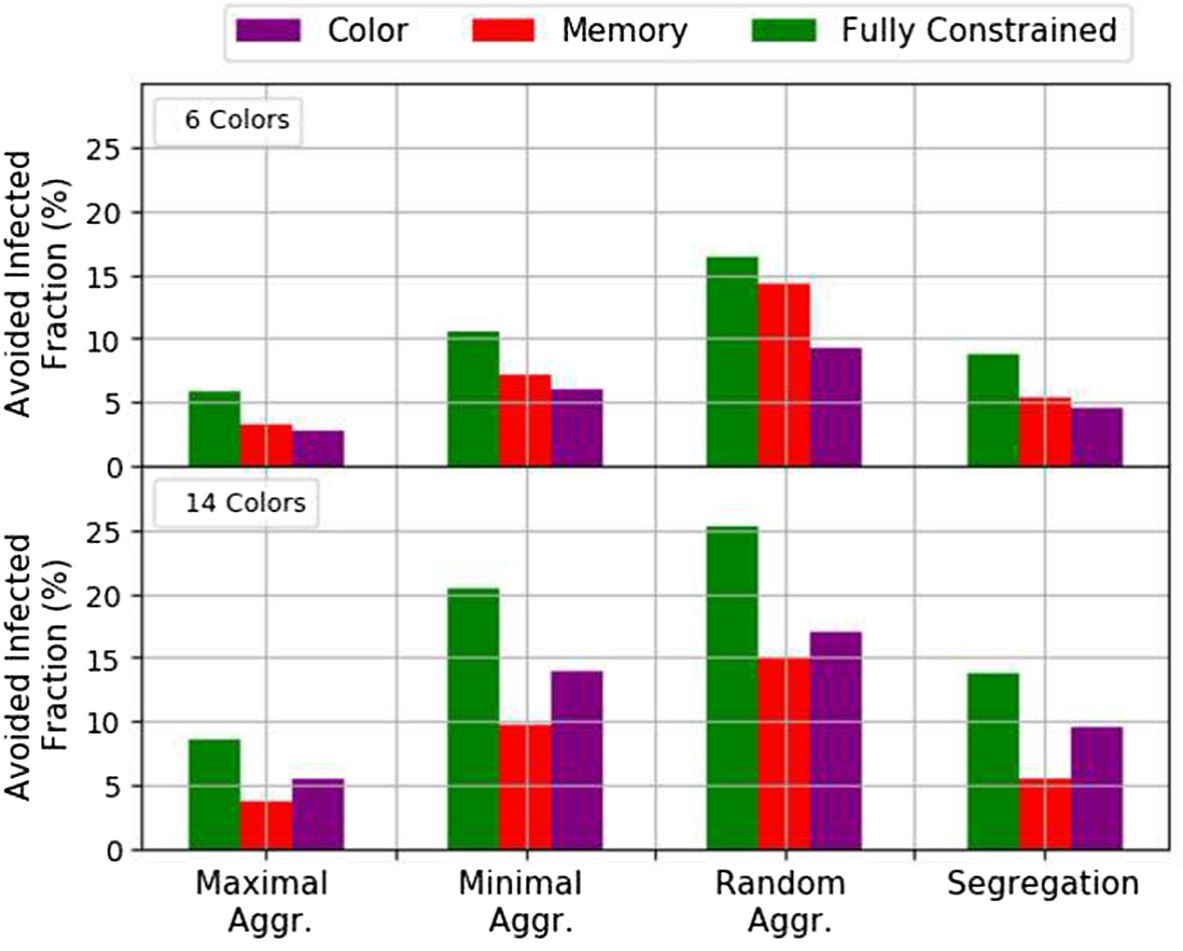
Fraction of avoided infected agents considering different forms of social interactions compared to the scenario of no memory and not respecting the colors for the original network. Top panel: strategies are with $N_c = 6$; bottom panel: $N_c = 14$. Error bars are not plotted for being negligible

We consider here a multiplex network formed by Work, Household, and Social layers. We simulate the epidemic spreading in this multiplex network by following a synchronous SIR-like process. To this aim, we consider the merged network composed by aggregating all layers onto a single one, where the infection probability $\beta$ is different for each layer. This choice reflects the idea that in different environments, the infection can be more or less probable upon contacts, because of different behaviors. For instance, at home people may be less prone to enforce social distancing and wear masks, with respect to work or social environments. This translates into considering the infection probability as a node variable: we assume the infection probability of node *i* in layer *L*, $\beta ^L_i$, to depend on the layer *L* and on the node’s degree in the corresponding layer, $\kappa ^L_i$ (Aleta et al. 2020; Perez et al. 2020). The infection probability is thus defined as $\beta ^L_i = \frac{\beta ^L}{\kappa ^L_i}$, where $\beta ^L$ is the infection probability in layer *L*. In particular, we choose $\beta ^{household}=0.50$, $\beta ^{work}=0.30$, $\beta ^{social}=0.20$, so that we assume, without loss of generality, $\sum _L \beta ^L = 1$. In this way, we assume the infection probability to be larger in households compared to work places, and even lower in social environment, as previously explained. Furthermore, we assume that the infection probability is inversely proportional to $\kappa ^L_i$. In this way, we consider that individual behavior changes as a function of the size of the gathering. The larger the gathering (e.g. in. the office or within a social context), the more people may be willing to enforce social distancing and wear masks. That is, we assume that the bigger the number of interacting people, the larger is the average distance among them (see for example Nunn et al. (2015)). Conversely, in small gatherings (e.g. at home) people may be less prone to change their behavior. Finally, we set $\gamma =0.30$ and the initial fraction of randomly chosen infected agents to be equal to $1\%$.

We start our exploration of the effect of different lockdown strategies without considering the Social layer, namely by setting $\beta ^{social}=0$. Figure 5 shows the final fraction of recovered individuals $R_\infty$, averaged over 1000 runs. As expected, the more colors the less infected individuals, due to a more disconnected network. With that in mind, the *Random Aggregation* strategy is the best to contain the spread of the epidemic, because of the smaller size of the largest connected component, followed by the *Minimal Aggregation* strategy, the *Segregation* strategy and finally the *Maximal Aggregation* strategy, in consonance with the results presented in Fig. 4. Note that these strategies are ranked precisely in the reverse order with respect to the number of conflicts $\chi$ (see Fig. 4).

## Effect of social interactions

We now explore the effects of social interactions, by adding the Social layer that accounts for all interactions that do not occur at work or in households. This layer is built as an Erdos Reny graph with average connectivity $\kappa _{social}$, in order to simulate (in a simplified way) the limitation of the number of contacts imposed by different NPIs. In other words, each node in the Social layer will randomly connect, on average, to a given number of nodes $\kappa _{social}$ assumed to be 4. In the Additional file 1: Sect. II, Fig. S3, we verified that for different values of $\kappa _{social}$ the results will not significantly change. Moreover, we considered four different situations about how the links can be formed in the Social layer. Links can indeed be added by respecting color (i.e. within the same partition), that is, two nodes can be connected in the Social layer only if they share the same color, or without respecting it. Furthermore, links can be generated with or without memory, that is, at every time step the links are rewired or not, respectively.

**Unconstrained (no memory, no color)**: the Social layer is time-dependent, changing at each time step, and links are randomly formed between nodes regardless of the color.
**Color**: the Social layer is time-dependent, changing at each time step, and links are randomly formed only between nodes of the same color.
**Memory**: the Social layer is static, without time-dependence, and links are randomly formed between nodes regardless of the color.
**Fully constrained (memory and color)**: the Social layer is static, without time-dependence, and links are randomly formed only between nodes of the same color.

From here on, we will include the Social layer in the multiplex representation and consider the four possible forms of social interactions described above. Of course, by increasing the overall degree of the network, we expect the epidemic outbreak to increase as well. However, our aim in this Section is to study how different kinds of social interactions with different degree of severity (with memory, respecting the partitions in different colors, etc.) impact the epidemic spreading with respect to the different partition strategies adopted.

First, we analyze the effects of including a Social layer whose interactions do not follow any restriction: the unconstrained case (no memory, no color). In Fig. 6 we compare the consequences of including the Social layer in the worst case scenario (unconstrained), compared to the original setting (without the Social layer) for the case of 10 colors. Fig. 6 shows that with the addition of the Social layer the unconstrained case results in a much larger fraction of infected individuals, regardless of the strategy employed for the network partition. We conclude that the inclusion of an unconstrained Social layer destroys the effectiveness of the colored strategies to contain the epidemic.

In the following we explore more restricted forms of social interactions. Figure 7 shows the fraction of avoided infections for the different forms of social interactions compared to the previously studied unconstrained situation, for a case with a small number of colors (6) and a case with a large number of colors (14). Several comments are in order. First, the greater the number of colors, the better the containment of the epidemic. Second, the best strategies are the ones that return a more disconnected network, namely the *Random Aggregation* and *Minimal Aggregation* strategy. Finally, for a small number of colors, forcing social interactions with memory (within a fixed set of individuals) is more effective than imposing colored social interactions (among individuals sharing the same color). At the opposite, for a larger number of colors, it is more effective to constrain social interactions within individuals sharing the same color instead of social interactions with memory. In both cases, fully constrained social interactions with both colors and memory is the most effective strategies to avoid additional infections.

In Table 1 we summarize the fraction of recovered individuals $R_{\infty }$ for different compositions of the multiplex network and with different constraints in building the Social layer. One can see that the implementation of a *Minimal Aggregation* partition strategy is extremely effective in reducing the impact of the disease, of about two thirds. At the same time, the economic burden of such strategy is relatively limited, with $14\%$ of individuals forced to stay home and temporarily lose their job. However, the inclusion of a Social layer dramatically alter this picture: the epidemic outbreak increases from $32\%$ to $81\%$ of the population. This is due to the fact that, in a network formed by interacting cliques, the presence of a small number of random interactions can dramatically worsen the effect of an epidemic. Finally, one can see that such epidemic outbreak can be reduced by imposing constraints to social interactions. The most effective one is the joint application of color and memory constraints in building the Social layer.

**Table 1:** Fraction of recovered agents $R_{\infty }$ for different compositions of the multiplex network: work and Household only, and with the inclusion of a Social layer

Network layers	Constraints	$R_{\infty }$ (%)
Work & Household	–	32
	Color (*Minimal Aggregation*)	12
Work & Household & Social	–	81
	Memory	78
	Color (*Minimal Aggregation*)	71
	Color (*Minimal Aggregation*) + Memory	64

We compare the cases with no restrictions and with different combinations of constraints. We consider here the *Minimal Aggregation* strategy with ten colors, whose fraction of conflicts is $\chi =14\%$

## Conclusions

In this work, we proposed a network approach to model the implementation of different strategies for a partial lockdown. Our model is composed by two main ingredients: a multiplex network including social interactions within different contexts, and numerical simulations of a SIR process to mimic the epidemic spreading. We proposed different strategies to segregate the network into disconnected components (partitions) with a twofold goal: halting the epidemic spreading, whose effectiveness can be measured by the reduction in the number of infected individuals, and minimizing the economic burden of the partial lockdown, that can be quantified by the removed links in the Work layer, that represent job losses. We found that the best partition strategy for containing the epidemic is a *Random Aggregation* strategy, but this comes with the larger job loss. A good compromise is the so-called *Minimal Aggregation* strategy, which is able to create a good segregation in the network while also minimizing the link removal. We showed that the inclusion of unconstrained social interactions dramatically increased the spreading of the disease. As a consequences, we studied different constraints to be applied specifically to the links in the Social layer: only within the same partition (joining nodes with the same color) and/or with memory (individuals interact with the same peers over time). With a number of color high enough, imposing color on the social interactions is more effective than maintaining memory, while clearly the two methods together would be the best combinations to reduce the epidemic outbreak.

Our work comes with limitations. For instance, we did not include schools in the network modelling, assuming that schools can stay temporarily closed during partial lockdowns. In future work, schools could be included in the model by adding another layer in the multiplex network, as in the reference Aleta et al. (2020). Another way of taking schools into account is to consider the work layer as a *work/school/nursing* layer, adjusting the group size distribution. Another limitation of our work is that the multiplex network that models social interactions is built on several assumptions and not directly by using empirical data regarding contact matrix within work or household contexts. However, data such as the precise composition of households or workplaces are not readily available. Furthermore, we adopted a very simple model for the epidemic spreading—the SIR model—, which is not realistic. This choice is however motivated to keep the number of parameters of the model, which is quite large, as low as possible. In future works, it would be interesting to explore the effects of the proposed partition strategies and restriction for social interactions on empirical data, regarding both the network reconstruction and the disease propagation. Finally, it would be interesting to include mobility data to test if the proposed partition strategies could be realistically implemented.

## Supplementary Information

**Additional file 1:** Supplementary Material.

## Data Availability

The data that support the findings of this study are available from the corresponding authors upon reasonable request.

## Abbreviations

SIR: Susceptible-Infected-Recovered
NPIs: Non-Pharmaceutical Interventions

## Footnotes

1 https://www.worldbank.org/en/news/press-release/2020/06/08/covid-19-to-plunge-global-economy-into-worst-recession-since-world-war-ii or a related post

2 Percentages obtained from the study of the Barcelona municipal register 2019: https://www.bcn.cat/estadistica/catala/dades/tpob/llars/padro/a2019/edat/t23.htm.
