# Supplementary material for: Modeling partial lockdowns in multiplex networks using partition strategies
Source: Appl Netw Sci. 2021 Apr 1;6(1):27. doi: 10.1007/s41109-021-00366-7 (PMC8012750; doi:10.1007/s41109-021-00366-7)
Supplement: Supplementary file 1 — Additional file 1: Supplementary Material. [file 41109_2021_366_MOESM1_ESM.pdf]

Supplementary Materials for the manuscript entitled:
Modeling partial lockdowns in multiplex networks using partition strategies

Adrià Plazas, Irene Malvestio, Michele Starnini & Albert Díaz-Guilera

I. DEPENDENCE ON THE WORKPLACE/HOUSEHOLD SIZE DISTRIBUTION

The household size distribution of our model is inferred from empirical data of the city of Barcelona. The distribution of company size instead is hypothetical. In this section, we aim at studying if some characteristics of household and company size distribution could affect the results of our paper. We therefore consider other realistic distributions.

In Figure S1 we show the effectiveness of the partition strategies (as assessed by the fraction of conflicts χ, the relative size of the biggest connected component G, and the entropy of the color distribution S, as a function of the number of colors in the network N_c) in dependence of the workplace size distribution. For simplicity, we choose all the distribution of workplace sizes as Gaussian with different means and standard deviations:

- Size 0: mean 15, standard deviation 5 (same as for the results shown in the main manuscript);
- Size 1: mean 20, standard deviation 5;
- Size 2: mean 25, standard deviation 5;
- Size 3: mean 20, standard deviation 10.

From the Figure S1 we conclude that the influence of the company size distribution is minimal. Figure S2 shows the effectiveness of the partition strategies in dependence of the household size distribution:

- Size 0: 1 member 10%, 2 : 80%, 3: 10%;
- Size 1: 1 member 38%, 2 : 38%, 3: 14%, 4: 8%, 5: 1.5%, 6: 0.5% (same as for the results shown in the main manuscript);
- Size 2: 1 member 23%, 2 : 28%, 3: 19%, 4: 13%, 5: 11.5%, 6: 5.5%;
- Size 3: 1 member 13%, 2 : 18%, 3: 19%, 4: 23%, 5: 16.5%, 6: 5.5%, 7: 5%.

These distributions are less skewed (Size 0) and more skewed (Size 2 and 3) versions of the original one. From the results in Figure S2 we notice that household distribution with higher percentage of densely populated households give rise to a higher number of conflicts and bigger connected components.

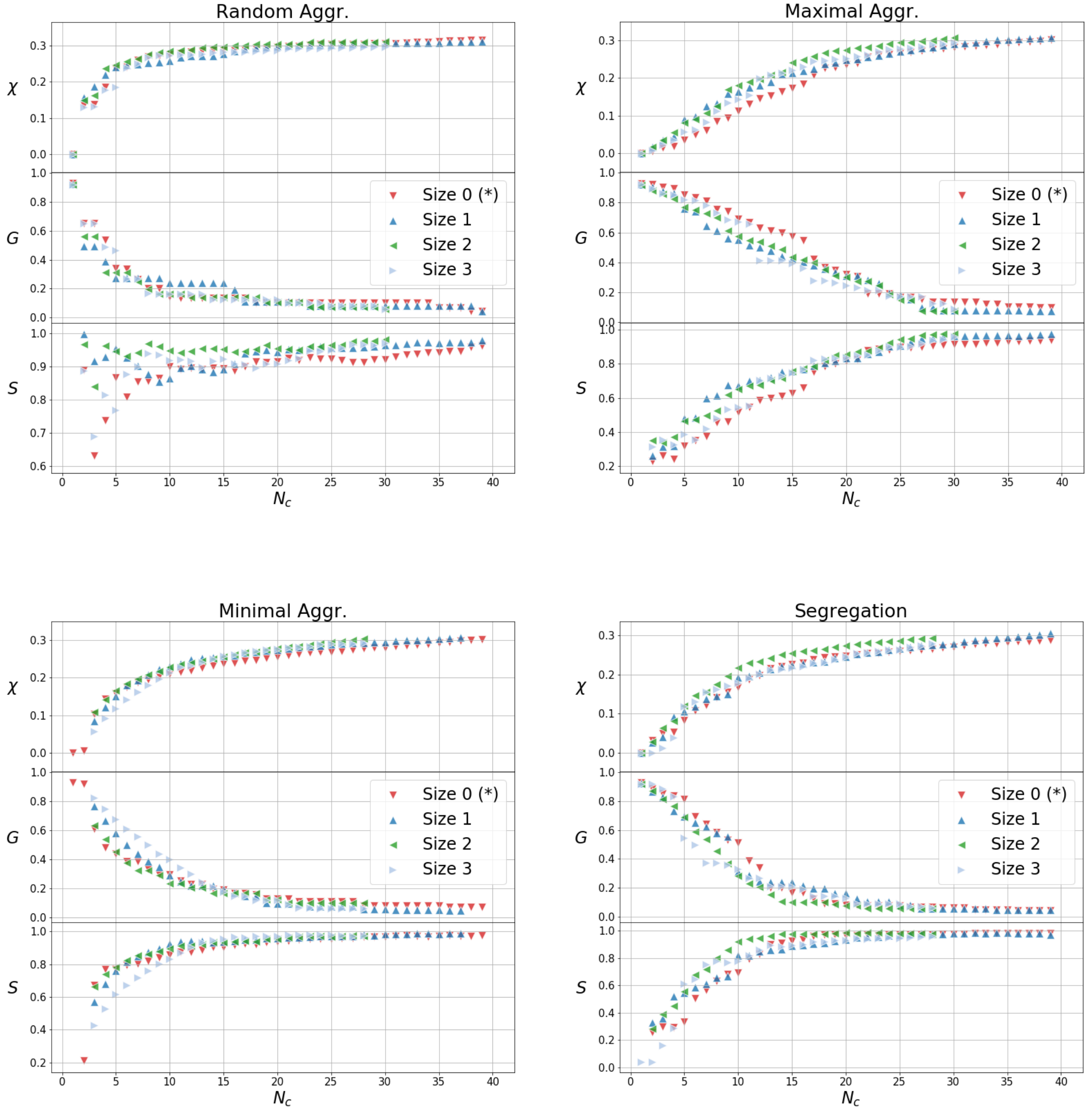

FIG. S1: Effectiveness of the partition strategies (*Random Aggregation*, *Maximal Aggregation*, *Minimal Aggregation*, *Segregation*) for different workplace size distributions. The number of nodes is $N_n = 1000$. The asterisk (Size 0) refers to the distribution that we use in the main analysis of the paper. We see how the influence of the company size distribution is minimal.

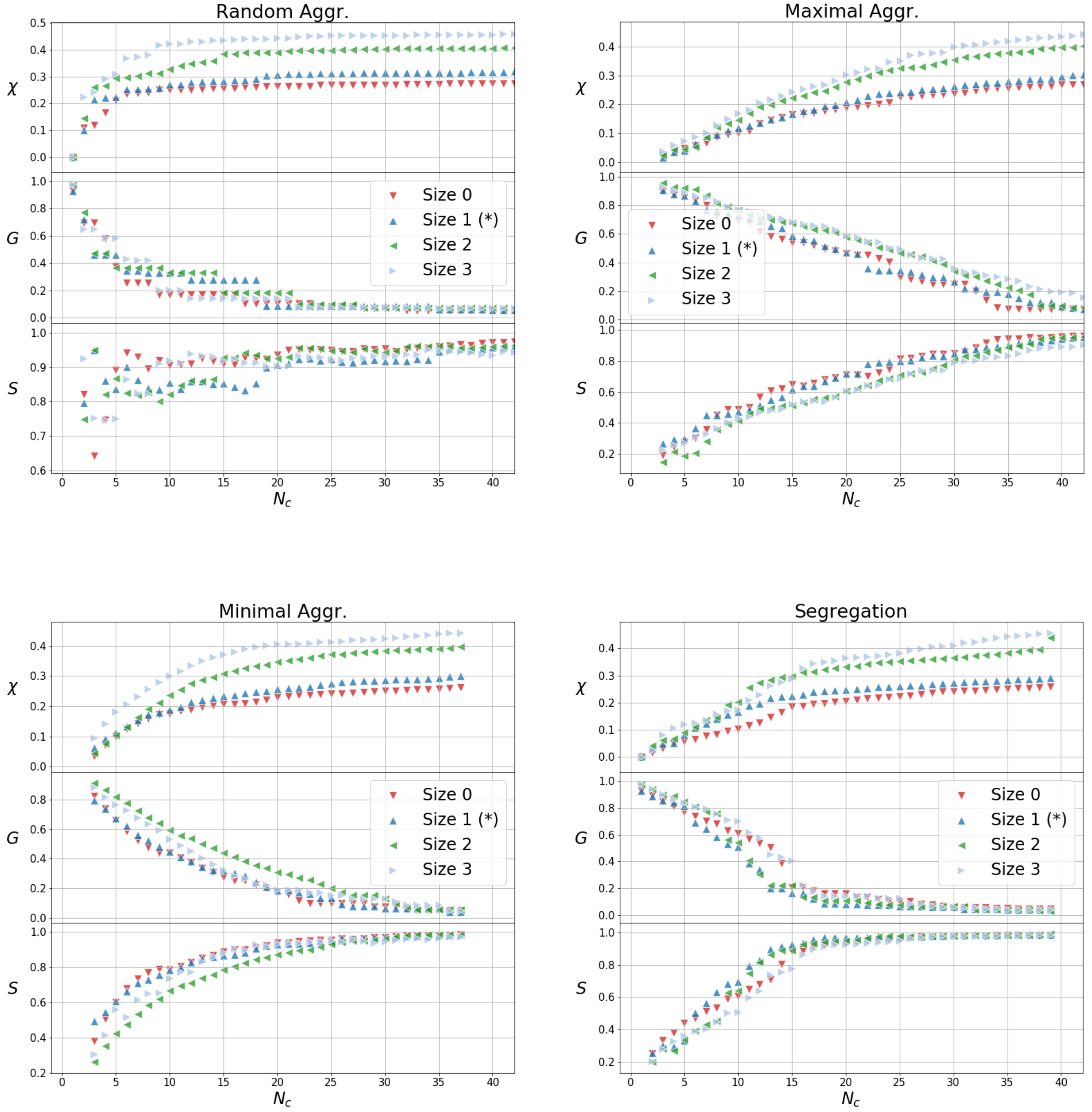

FIG. S2: Analogous to the previous Figure, here in dependence of the household size distributions. The number of nodes is $N_n = 1000$. The asterisk (Size 1) refers to the household size distribution that we use in the main analysis of the paper. We notice that households with more members give rise to a higher number of conflicts and bigger connected components.

II. DEPENDENCE ON THE SOCIAL LAYER TOPOLOGY

In order to study the effectiveness of the proposed lockdown strategies to different number of random contacts we have simulated the cases of an Erdo Reny with $\kappa_{social} = 2$ and $\kappa_{social} = 8$ As can be seen in S3 the different number of random contacts of the Social Layer does not significantly modify the effectiveness of the strategies (compared to the case presented in the main text), apart from the fact that the greater the connectivity in the social layer, the greater difficulty in containing the epidemic. That is to say that the presented conclusions in the main text remain valid:

- The greater the number of colors, the better the containment of the epidemic
- The best containment strategies are the Minimal Aggregation and the Random Aggregation.
- Fully constrained social interactions is the most suitable form of social interaction for avoiding infections. Also, for small number of colors it's better to keep always the same contacts, namely the Memory of the Social Interaction. However, for a large number of colors it's better to keep the color than the memory while interacting, though this not always happens in the case of $\kappa_{social} = 2$

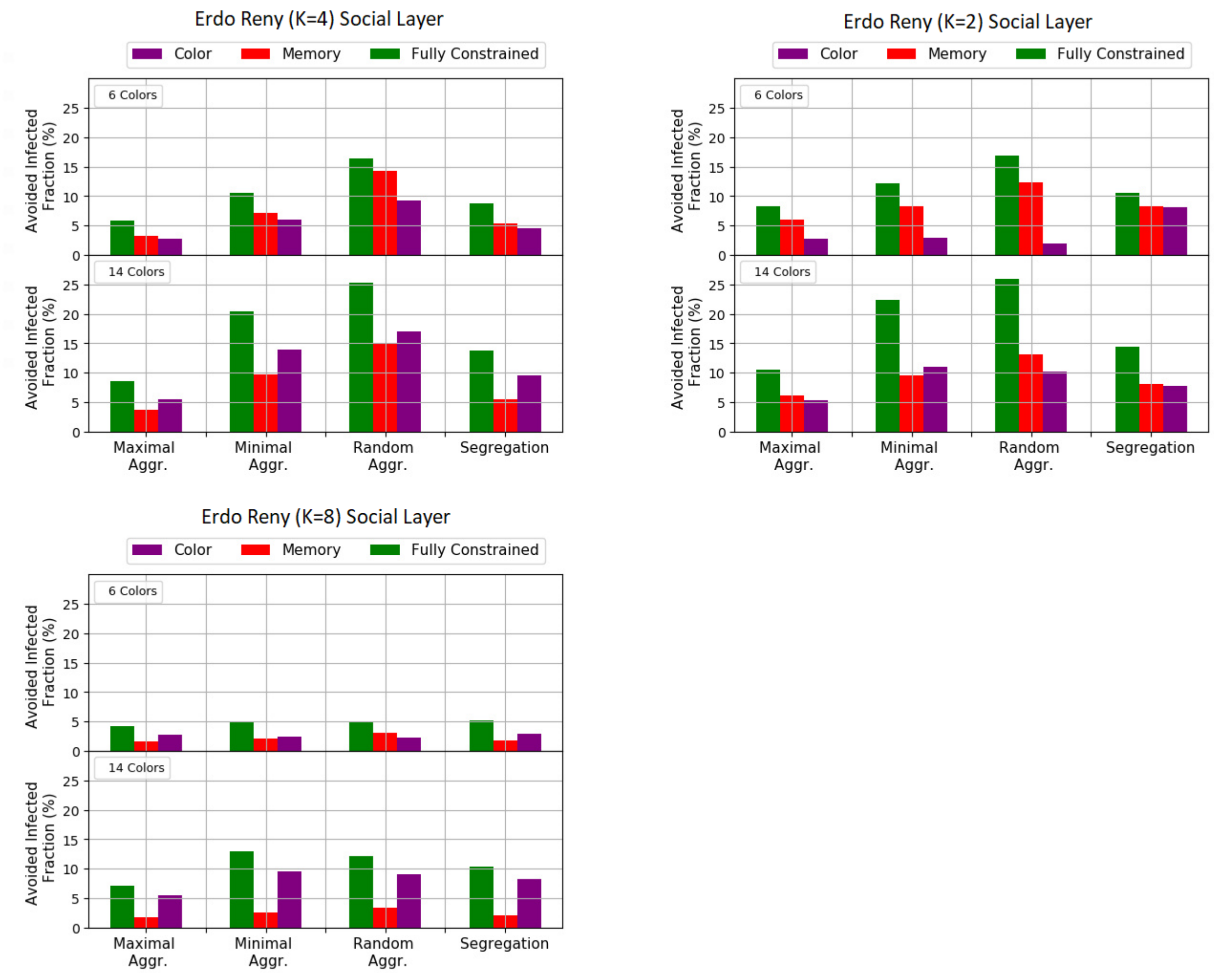

FIG. S3: Fraction of avoided infected agents for different number of random contacts considering different forms of social interactions compared to the scenario of no memory and not respecting the colors for the original network. Top panel: strategies are with $N_c = 6$; bottom panel: $N_c = 14$. Error bars are not plotted for being negligible.
